# Variation, Overlap, and Stability in Defining Safety Net Hospitals

**DOI:** 10.1001/jamanetworkopen.2025.23923

**Published:** 2025-07-30

**Authors:** Paula Chatterjee, Joshua M. Liao, Kano Amagai, Yueming Zhao, Torrey Shirk, Amol S. Navathe

**Affiliations:** 1 Department of Medicine, Perelman School of Medicine, University of Pennsylvania, Philadelphia; 2 Department of Medical Ethics & Health Policy, Perelman School of Medicine, University of Pennsylvania, Philadelphia; 3 Division of General Internal Medicine, University of Texas Southwestern Medical Center, Dallas; 4 Department of Medicine, Penn Presbyterian Medical Center, Philadelphia, Pennsylvania; 5 Leonard Davis Institute of Health Economics, University of Pennsylvania, Philadelphia

## Abstract

**Importance:**

The lack of universally accepted definitions for safety net hospitals (SNHs) has made it difficult to effectively design policies to support these hospitals and the populations they serve.

**Objective:**

To evaluate the overlap, variation, and consistency across different definitions for SNH status.

**Design, Setting, and Participants:**

This retrospective cohort study used a hospital year–level dataset on short-term acute care US hospitals from 2014 to 2022. Hospital-level and area-level measures were used to define SNHs. Hospital characteristics under each definition, overlap across definitions, and stability of SNH samples produced by each definition from were described. Data analyses were performed from August 2024 to June 2025.

**Exposure:**

Nine hospital-level and 4 area-level SNH definitions.

**Main Outcomes and Measures:**

Hospital characteristics under each definition, overlap across definitions, and stability of SNH samples over time. Hospital-level definitions included Medicare Disproportionate Share Hospital (DSH) index, Medicare inpatient day share, dual-eligible or low-income subsidy (DLIS) inpatient day share, Medicaid inpatient day share, Medicare Safety-Net Index, teaching status, public ownership, uncompensated care share, and operating margins. Area-level measures included Area Deprivation Index, Social Vulnerability index, proportion Hispanic population, and proportion Black population. Safety net status was assigned based on quartiles defined nationally (or within a state for Medicaid-specific definitions). For a subset of measures, this quartile-based approach was compared between the absolute number of inpatient days attributed to each patient group and the relative number (or share) of inpatient days.

**Results:**

Among 4531 short-term acute care hospitals, between 992 (21.9%) and 1326 (29.3%) were SNHs in 2022, depending on definition. SNHs defined based on the absolute level of inpatient days or absolute level of DLIS populations were often large (51% [242 of 476] or 67% [537 of 801]) and were not often rural (9% [45 of 476] or 2% [17 of 801]). Meanwhile, SNHs defined based on relative level of Medicaid inpatient days or relative level of DLIS patients were more often small (63% [298 of 476] and 82% [660 of 801]) and rural (48% [228 of 476] and 69% [555 of 801]) hospitals. The largest overlap across definitions was between a hospital’s Medicaid inpatient day share and Medicare DSH index (55% overlap [808 of 1466 hospitals]), which tended to represent large, teaching hospitals. Public ownership, teaching status, and Medicare DSH index produced the most stable definitions of SNHs over time from 2014 to 2022, with 83% (862 of 1043), 74% (1000 of 1354), and 60% (809 of 1358) of similar hospitals, respectively, meeting safety net criteria. The least stable definitions were based on low operating margins, high uncompensated care share, and high DLIS day share, with only 15% (263 of 1796), 20% (362 of 1823), and 25% (436 of 1725) of similar hospitals, respectively, meeting safety net criteria in 2014, 2018, and 2022.

**Conclusions and Relevance:**

In this cohort study of US hospitals, different SNH definitions produced different samples, and candidate measures had variable overlap and stability over time. These findings highlight the trade-offs when considering different options to define SNHs.

## Key Points

**Question:**

To what extent do different definitions for safety net hospitals overlap, change over time, and represent similar types of facilities?

**Findings:**

In this cohort study of 4531 short-term acute care hospitals, between 1% and 55% of similar hospitals were represented across pairs of definitions, with some definitions producing different samples of hospitals year to year. Different definitions produced different groups of hospitals over time, risking overinclusion and/or underinclusion of certain hospitals across different payment programs.

**Meaning:**

The findings highlight the trade-offs when considering options to define safety net hospitals.

## Teaser

This cohort study examines hospital-level and area-level measures for defining safety net hospitals to assess for overlap, variation, and consistency among the different definitions of safety net status.

## Introduction

Safety net hospitals (SNHs) care for low-income, uninsured, and underinsured populations in the US.^1^ Despite this critical role of serving patients who might otherwise not be able to access care, SNHs remain a largely undefined group of organizations.^2^ The lack of a universally accepted definition for SNHs has made it difficult to design and target policies to support their mission,^3^ and the existence of multiple definitions can also introduce policy challenges. First, the risk of underinclusion or overinclusion of hospitals under different definitions can threaten the efficient allocation of local, state, and federal resources. To date, state and federal subsidy programs intended to support SNHs often end up reaching hospitals with healthy financial margins that provide little service to safety net populations.^4^ Second, multiple definitions can make it difficult to target and deploy resources to SNHs in emergent situations such as pandemics, natural disasters, or cybersecurity threats, which have become increasingly common in recent years.^5,6,7^ For these reasons, formal definitions for SNHs are needed, but there is little evidence to guide these efforts.^8^

Despite several candidate options, there is little consensus on how SNHs should be defined,^9^ and different hospitals may be represented under different definitions.^10,11^ Some measures focus on hospital characteristics, such as the share of care provided to patients with public insurance or a hospital’s public ownership status. Other measures are based on area-level socioeconomic measures, such as social disadvantage indices, to capture hospitals’ broader service and proximity to marginalized communities. To incorporate goals of population health equity, some have argued that SNH definitions should incorporate the share of racial and ethnic minority groups in surrounding communities to better allocate health care resources to areas of historical underinvestment.^12^ Even after a definition is chosen, there is little consensus on whether absolute or relative levels of safety net service should be used: should SNHs provide the most care to low-income populations overall or to low-income populations relative to others? While there is no gold standard, it is critical for policymakers to understand the risks and trade-offs across different definitions to design policies that support care delivery for low-income populations.^3^

In this study, we evaluated overlap, variation, and consistency across different candidate definitions for SNH status. We evaluated both hospital- and area-level measures and examined the extent to which assignments of safety net status are stable over time. We also examined the implications of absolute vs relative thresholds of safety net clinical service and described the extent to which samples of SNHs differ across these dimensions. This study adds to existing literature by highlighting the trade-offs of different definitions, evaluating overlap and potential redundancy across definitions, and characterizing stability in definitions over time to guide state and federal decisions on resource stewardship.

## Methods

### Data and Variables

We used a hospital year–level dataset from 2014 to 2022. We obtained annual information on hospital characteristics (number of beds, teaching status, public ownership, rurality, and critical access hospital status) and finances (Medicare Disproportionate Share Hospital [DSH] index, Medicare inpatient day share, Medicaid inpatient day share, operating margins, and uncompensated care share out of total operating expenses) from the Healthcare Cost Reporting Information System (HCRIS) compiled by RAND.^13^ This cohort study was approved by the institutional review board at The Perelman School of Medicine, University of Pennsylvania, which waived the informed consent requirement because this was not a human participants research due to use of hospital-level data. We followed the Strengthening the Reporting of Observational Studies in Epidemiology (STROBE) reporting guideline.

We used the 100% of the Medicare Provider Analysis and Review (MedPAR) file, 100% of inpatient Medicare fee-for-service (FFS) claims, a random 20% sample of outpatient Medicare FFS claims, and the 2014 to 2022 Denominator File to calculate a hospital’s annual Medicare Safety-Net Index (MSNI). The MSNI uses 3 measures to capture safety net service: (1) the share of Medicare volume attributable to beneficiaries receiving Medicare Part D’s low-income subsidy, (2) revenue share associated with uncompensated care, and (3) share of total volume attributable to Medicare beneficiaries.^14^ We also used inpatient Medicare FFS claims, MedPAR, HCRIS, and the Denominator File to calculate a hospital’s share of inpatient days for DLIS beneficiaries—those dually eligible (for both Medicare and Medicaid) or who qualified for the Part D low-income subsidy—out of total inpatient days.

To assign area-level measures of SNH status, we geocoded hospitals by their physical addresses and linked to different area-level units. From the American Community Survey 5-year estimates (with terminal years overlapping with the study period),^15^ we obtained zip code–level estimates for the proportion of population that self-identified as Black or Hispanic individuals and linked to hospital zip codes based on hospitals’ physical addresses. We collected racial and ethnic demographic data because minority populations are more likely to be uninsured, underinsured, and insured by public insurance programs and, therefore, more likely to be served by SNHs.^16^

From the Neighborhood Atlas, we obtained Census block group estimates of the Area Deprivation Index (ADI), which has been used by the Centers for Medicare and Medicaid Services to identify safety net populations.^17^ From the Center for Disease Control and Prevention, we obtained Census tract–level estimates of the Social Vulnerability Index (SVI), which has been used in government programs to capture social disadvantage and allocate resources.^18^ These indexes were linked to the corresponding Census blocks and tracts associated with hospitals’ physical addresses.

### Statistical Analysis

We chose 9 hospital-level and 4 area-level measures to define SNHs. These measures were chosen based on review of literature and existing policy proposals and in alignment with stated policy goals of population health equity from public and private payers (eTable 1 in Supplement 1).^9,11,19,20,21,22,23^

Hospital-level definitions included hospitals in the top quartile nationally for Medicare DSH index, top quartile nationally for Medicare inpatient day share, top quartile nationally for MSNI, top quartile nationally for DLIS inpatient day share, top state-specific quartile for Medicaid inpatient day share, bottom quartile nationally in operating margins, and top state-specific quartile for uncompensated care share; teaching hospitals; and hospitals operating under public ownership. The choice of using state or national distributions to determine quartiles was based on the extent to which the Medicaid program was part of the measure.

Among area-level definitions, we identified hospitals in the top quartile nationally by the proportion of population that identified as Black individuals and in the top quartile nationally by the proportion of the population that identified as Hispanic individuals based on the demographics of the hospital’s zip code. We identified hospitals in the top quartile nationally (highest disadvantage) for the ADI based on the hospital’s Census block group and in the top quartile nationally (highest disadvantage) for SVI based on the hospital’s Census tract.

We compared hospital characteristics across samples of SNHs under each definition. Comparisons were made separately for hospital- and area-level measures. Statistical significance was not assessed since formal hypotheses were not tested.

We determined the degree of overlap across safety net definitions. We identified the number and percentage of hospitals that qualified for safety net status in all possible pairs of definitions.

Next, we evaluated the extent to which each definition produced a stable set of SNHs over time. Understanding whether the composition of the safety net changed over time can inform short-term and long-term budgetary decisions by states and federal governments. For example, California and New York have recently considered new state-level funding pools to support SNHs and other financially distressed hospitals.^24,25^ Characterizing the extent to which SNH samples fluctuate from year to year can inform the size and design of such funding sources. To evaluate stability, we calculated the number of hospitals that met safety net status in any 1 of 3 years: 2014, 2018, and 2022. We chose these years to maximize completeness in reporting the most recent data available in HCRIS. We then calculated the number and proportion of hospitals that met safety net status in all 3 of these years, 2 of 3 years, and 1 of 3 years. We repeated this analysis for each hospital- and area-level definition.

We evaluated 2 approaches to establishing thresholds for assigning safety net status. The purpose was to evaluate definitions of SNH status based on whether a hospital provided most of the care for low-income populations broadly or as an organization. The first approach was to define quartiles of the hospital distribution based on the absolute number of Medicare inpatient days, Medicaid inpatient days, or DLIS inpatient days attributed to the hospital. The second approach was to define quartiles of the hospital distribution based on the relative number (or share) of Medicare inpatient days out of total inpatient days, Medicaid inpatient days out of total inpatient days, and DLIS inpatient days out of total inpatient days attributed to the hospital.

We tested these approaches using 3 definitions—Medicare inpatient days, Medicaid inpatient days, and DLIS inpatient days—that captured different dimensions of safety net care and were salient to the primary payers for low-income populations. For each measure, we compared hospital characteristics between SNHs based on their high absolute or share of care for Medicaid, Medicare, and DLIS patients. Again, statistical significance was not evaluated since formal hypotheses were not tested. All analyses were performed from August 2024 to June 2025 using SAS (SAS Institute) and R 4.3.1 (R Project for Statistical Computing).

## Results

Descriptive statistics for each definition are available in eTable 2 in Supplement 1. There were 4531 short-term, acute care hospitals in the national sample in 2022 (eTable 2 in Supplement 1). Of these hospitals, between 992 (21.9%) and 1326 (29.3%) were SNHs across hospital-level definitions, and between 1057 (23.3%) and 1117 (24.7%) were SNHs across area-level definitions (eTables 3 and 4 in Supplement 1). Sample differences reflected missingness, with the greatest missingness observed among 1659 hospitals missing ADI data and 1472 hospitals missing SVI data in 2022.

### Characteristics of SNHs Across Definitions

SNHs defined using Medicare DSH index and Medicaid inpatient day share were similar in size (353 of 1133 hospitals [31%] in the top Medicare DSH quartile and 330 of 1141 [29%] in the top Medicaid inpatient day share quartile hospitals had greater than 300 beds), teaching status (621 hospitals [55%] and 576 hospitals [50%]), and mean (SD) operating margins (0.0 [0.01] and 0.0 [0.01]) (eTable 3 in Supplement 1). SNHs defined using public ownership status were similar to those defined using uncompensated care shares: both groups were often small (761 of 992 public hospitals [77%] and 768 of 1141 top uncompensated care share hospitals [67%] had 0-99 beds) and located in nonmetropolitan areas (644 public hospitals [65%] and 551 of top uncompensated care share hospitals [48%]). SNHs defined using ADI were more often located in areas with smaller mean (SD) proportions of Hispanic populations than those defined using top quartile SVI hospitals (10.4% [15.0%] vs 20.8% [22.0%]) and were more often located in nonmetropolitan areas (703 of 1117 hospitals [63%] vs 428 of 1057 hospitals [40%]) (eTable 4 in Supplement 1).

### Overlap Across Safety Net Definitions

Overlap between pairs of SNH definitions ranged from 1% to 55% (Figure). Among hospital-level definitions, the largest overlap was between a hospital’s Medicaid inpatient day share and Medicare DSH index (808 of 1466 SNHs [55%] overlapped across definitions based on these 2 measures). There was comparatively more overlap between definitions of teaching hospital status and Medicare DSH index (34% [621 of 1838 hospitals]), MSNI and DLIS inpatient day share (34% [578 of 1825 hospitals]), and teaching hospital status and Medicaid inpatient day share (30% [576 of 1891 hospitals]). The smallest overlap was between Medicare inpatient day share and Medicaid inpatient day share (1% [20 of 2254 hospitals]), Medicare DSH index and Medicare inpatient day share (1% [30 of 2236 hospitals]), Medicare inpatient day share and teaching hospital status (7% [165 of 2294 hospitals]), and public hospital ownership and teaching hospital status (8% [167 of 2151 hospitals]).

**Figure.**
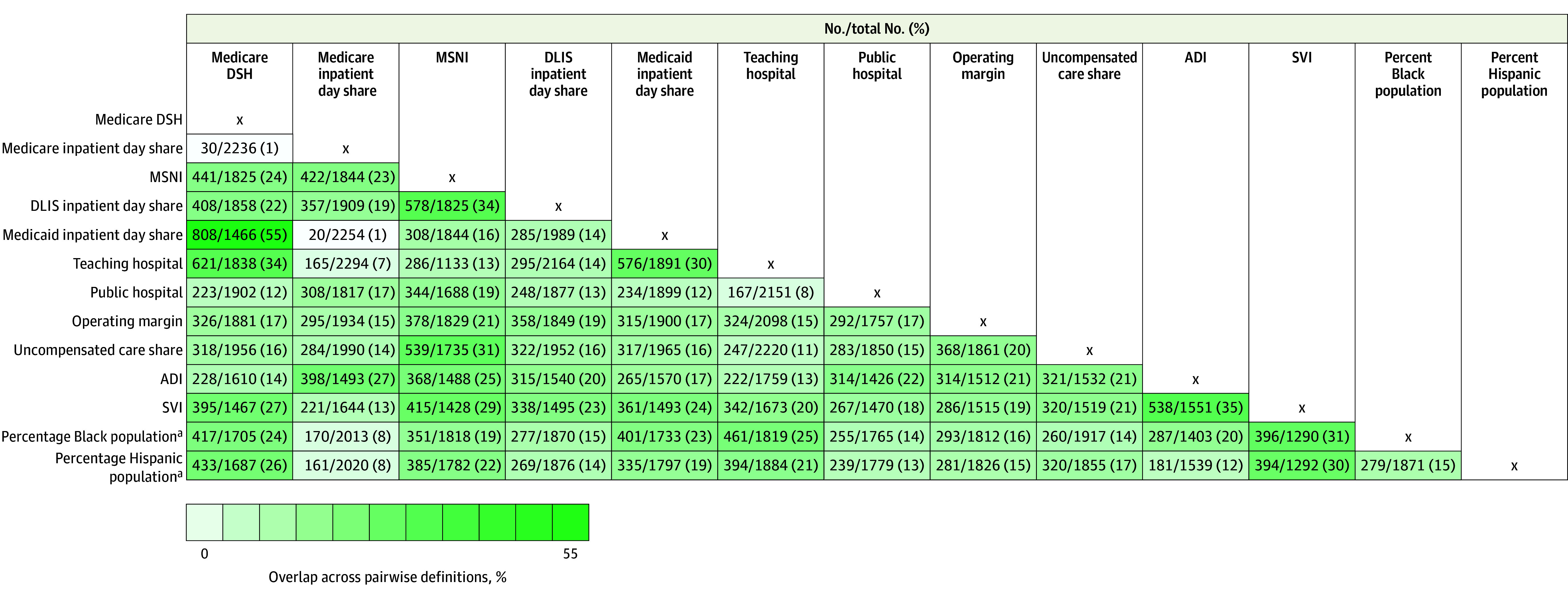
Pairwise Overlap Across Safety Net Hospital (SNH) Definitions Percentages overlap across pairwise definitions. ADI indicates Area Deprivation Index; DLIS, dual-eligible or low-income subsidy; DSH, Disproportionate Share Hospital; MSNI, Medicare Safety-Net Index; SVI, Social Vulnerability Index. ^a^Racial and ethnic minority populations were included because they are more likely to be uninsured, underinsured, or insured by public insurance programs and therefore more likely to be served by SNHs.

Among area-level definitions, the largest overlap was between SVI and ADI measures, which produced 35% overlap (538 of 1551 hospitals). The smallest overlap was between zip code–level measures of proportion of Hispanic population and ADI (12% [181 of 1539 hospitals]) and proportions of Hispanic and Black populations (15% [279 of 1871 hospitals]).

### Stability of Safety Net Status Over Time Across Definitions

SNHs defined using public ownership, teaching status, and Medicare DSH index produced the most stable samples over time, with 83% (862 of 1043), 74% (1000 of 1354), and 60% (809 of 1358) of similar hospitals meeting safety net criteria in 2014, 2018, and 2022, respectively (Table 1). The least stable definitions were low operating margins, high uncompensated care share, and high DLIS day share, which produced samples of SNHs in which only 15% (263 of 1796), 20% (362 of 1823), and 25% (436 of 1725) of similar hospitals meeting safety net criteria in 2014, 2018, and 2022, respectively.

**Table 1.** Stability of Safety Net Status Over Time Across Measures

Measure	SNH, No./total No. (%)		
	All 3 y	In 2 of 3 y	In 1 of 3 y
Hospital-level			
Medicare DSH	809/1358 (60)	262/1358 (19)	287/1358 (21)
Medicare inpatient day share	624/1504 (41)	396/1504 (26)	484/1504 (32)
MSNI	673/1411 (48)	323/1411 (23)	415/1411 (29)
DLIS inpatient day share	436/1725 (25)	514/1725 (30)	775/1725 (45)
Medicaid inpatient day share	719/1499 (48)	341/1499 (23)	439/1499 (29)
Teaching hospital	1000/1354 (74)	180/1354 (13)	174/1354 (13)
Public hospital	862/1043 (83)	87/1043 (8)	94/1043 (9)
Operating margin	263/1796 (15)	558/1796 (31)	975/1796 (54)
Uncompensated care share	362/1823 (20)	560/1823 (31)	901/1823 (49)
Area-level			
ADI	785/1175 (67)	148/1175 (13)	242/1175 (21)
SVI	645/1291 (50)	294/1291 (23)	352/1291 (27)
Proportion of Black population	888/1084 (82)	101/1084 (9)	95/1084 (9)
Proportion of Hispanic population	864/1067 (81)	107/1067 (10)	96/1067 (9)

Abbreviations: ADI, Area Deprivation Index; DLIS, dual-eligible or low-income subsidy; DSH, Disproportionate Share Hospital; MSNI, Medicare Safety Net Index; SNH, safety net hospital; SVI, Social Vulnerability Index.

Area-level safety net definitions were more stable over time. Proportion of Black population, proportion of Hispanic population, and ADI produced samples of SNHs in which 82% (888 of 1084), 81% (864 of 1067), and 67% (785 of 1175) of similar hospitals met safety net criteria in 2014, 2018, and 2022, respectively. The least stable area-level definitions was SVI, among which only 50% (645 of 1291) of similar hospitals met the safety net criteria in 2014, 2018, and 2022, respectively. Similar patterns were observed when comparing different combinations of the 3 chosen years (eTable 5 in Supplement 1).

### Comparison of Relative vs Absolute Thresholds Across Safety Net Definitions

Using Medicare inpatient day shares, we defined 3 groups of SNHs: 992 hospitals were among the top quartile of absolute Medicare inpatient days nationally, 992 hospitals were among the top quartile of relative level of Medicare inpatient days, and 141 hospitals were both among the top quartile of absolute and relative levels of Medicare inpatient days (Table 2). Hospital characteristics differed across these groups. SNHs in the top quartile of total Medicare inpatient days were often large (60% [594 of 992] with at least 300 beds), less often critical access hospitals (0), and less often in rural areas (3% [28 of 992]). SNHs in the top quartile of relative level of Medicare inpatient days were more often small (89% [882 of 992] with between 0-99 beds) and located in rural areas (67% [667 of 992]). SNHs that were in both absolute and relative groups were less often in rural areas (4% [6 of 141]) and more often medium-sized (71% [100 of 141] with 100-299 beds).

**Table 2.** Variation in Hospital Characteristics Across Relative (Share) and Absolute Levels of Safety Net Service in 2022

Characteristic	Levels of safety net service, No. (%)											
	Medicare inpatient days				Medicaid inpatient days				DLIS inpatient days			
	Non-SNH (n = 2406)	Relative only (n = 992)	Absolute only (n = 992)	Absolute and relative (n = 141)	Non-SNHs (n = 2914)	Relative only (n = 476)	Absolute only (n = 476)	Absolute and relative (n = 665)	Non-SNHs (n = 2597)	Relative only (n = 801)	Absolute only (n = 801)	Absolute and relative (n = 332)
Rural	1136 (47)	667 (67)	28 (3)	6 (4)	1465 (50)	228 (48)	45 (9)	99 (15)	1221 (47)	555 (69)	17 (2)	44 (13)
Ownership												
For-profit	487 (20)	127 (13)	146 (15)	28 (20)	502 (17)	111 (23)	70 (15)	105 (16)	494 (19)	119 (15)	116 (14)	59 (18)
Nonprofit	1362 (57)	559 (56)	719 (72)	111 (79)	1704 (58)	257 (54)	356 (75)	434 (65)	1485 (57)	455 (57)	559 (70)	252 (76)
Public	557 (23)	306 (31)	127 (13)	2 (1)	708 (24)	108 (23)	50 (11)	126 (19)	618 (24)	227 (28)	126 (16)	21 (6)
No. of beds												
0-99	1675 (70)	882 (89)	0	1 (1)	2151 (74)	298 (63)	26 (5)	83 (12)	1889 (73)	660 (82)	0	9 (3)
100-299	660 (27)	99 (10)	397 (40)	100 (71)	622 (21)	171 (36)	206 (43)	257 (39)	620 (24)	135 (17)	263 (33)	238 (72)
≥300	26 (1)	1 (0)	594 (60)	40 (28)	89 (3)	5 (1)	242 (51)	325 (49)	40 (2)	0	537 (67)	84 (25)
Critical access hospital	745 (31)	610 (61)	0	0	1264 (43)	63 (13)	3 (1)	25 (4)	1075 (41)	280 (35)	0	0
Share of total inpatient days, %^a^	8	2	33	4	4	1	7	14	1	1	5	2

Abbreviations: DLIS, dual-eligible or low-income subsidy; SNH, safety net hospital.

^a^ This row reflects the share of total inpatient days provided by SNHs in a given category. For example, in the first column, 8% reflects the fact that non-SNHs (as defined using Medicare inpatient days) provided 8% of inpatient days to all Medicare beneficiaries in 2022.

There were similar patterns in absolute vs relative comparisons using Medicaid inpatient days and DLIS admissions (Table 2). SNHs defined as those in the top quartile of absolute level of Medicaid inpatient days or absolute level of DLIS admissions were large (51% [242 of 476] and 67% [537 of 801]) and nonrural (9% [45 of 476] and 2% [17 of 801]). SNHs defined as those in the top quartile of relative level of Medicaid inpatient day or relative level of DLIS admission were more often small (63% [298 of 476] and 82% [660 of 801], respectively) and rural (48% [228 of 476] and 69% [555 of 801]). Overlap across high-absolute and high-relative SNHs was greatest for the measure of Medicaid inpatient days.

## Discussion

This national cohort study demonstrates variation across different definitions of SNHs. Measures varied in their degree of overlap and consistency over time. Even within measures, there was variation according to absolute levels of service provided to low-income populations vs the relative level (share) of safety net service provided by a given hospital.

These findings highlight that definitional choices matter, and transparent policy goals must pair with the choice of definition. For example, if the policy goal is to bolster the financial viability of struggling hospitals, then policymakers should consider identifying hospitals based on uncompensated care share and accept the limitation that the sample of hospitals may be less stable and more likely to fluctuate from year to year. If the policy goal is to encourage service delivery to low-income patient populations and target hospitals that do so year after year, then policymakers should define SNHs using teaching status or Medicaid inpatient day share. If the policy goal is to prioritize equity in resource allocation among hospitals that serve racially and ethnically minoritized groups, then safety net measures should include the racial and ethnic demographics of patients served. We offer these findings as context for policymakers to engage in transparent discussion about trade-offs in exchange for different policy goals.

The issue of SNH definitions has persisted, in part, because policy goals vary across payers. Different payers are responsible for different low-income populations, leading each payer to define the safety net for their own purposes. Medicare created the MSNI to focus on low-income older adults and those qualifying for low-income subsidies.^14^ Medicaid has its own safety net definitions that focus on Medicaid enrollees.^26^ Although unintentional, this fragmentation can play a role in redundancy across policy efforts. Several payment programs seek to financially bolster SNHs, including, but not limited to, Medicare DSH, Medicaid DSH, Upper Payment Limit Supplemental Payments, and the 340B Drug Pricing Program. However, despite growth in these programs, funding is often mistargeted and SNHs persist in financial precarity.^4,27,28,29^ Aligning population health priorities across payers is an important first step toward addressing these mistargeting challenges.

Policymakers must also decide whether safety net policies should target patients or the organizations that serve them. Most safety net populations receive care at hospitals that serve more patients overall (ie, larger hospitals in populous, urban areas). Designing policies that link safety net funding to high-absolute levels of care for low-income patients may be effective at promoting access; however, this approach may produce geographic trade-offs in that high Medicare, Medicaid, and DLIS share hospitals are more often rural hospitals that are increasingly serving populations with higher rates of poverty and with financial viability.^30,31^ These dynamics further underscore the need for clarity about policy goals in selecting safety net definitions.

### Limitations

This study has limitations. First, our safety net measures may reveal different patterns in different sites of care delivery (such as outpatient or postacute care). Second, we focused on commonly used hospital- and area-level definitions but may have excluded other important measures. Third, the use of zip code–level disadvantage indexes to accurately map catchment areas of hospitals^32^ may be prone to measurement error for large, tertiary and quaternary hospitals that attract patients from wider geographic areas.^33^ Fourth, privatization of public hospitals over the study period may have led to greater concentration of safety net care, particularly for patients with Medicaid insurance.^34^ Fifth, while we characterized the overlap across definitions, we were unable to determine how much this overlap reflects redundancy vs effective targeting under different policy objectives.

## Conclusions

This study highlights trade-offs when considering different options to define SNHs. These trade-offs range from the types of hospitals represented, stability of different measures over time, and the need to consider absolute vs relative levels of safety net service. Transparency about trade-offs and clarity about the connection between policy goals and definitions can inform policymakers seeking to support SNHs.

## Notes

Supplement 1. **eTable 1.** Description of Safety-Net Hospital Definitions & Implications for Policy and Payment **eTable 2.** Distribution of Safety-Net Candidate Measures, 2022 **eTable 3.** Characteristics of Safety-Net Hospitals Using Hospital-Level Definitions, 2022 **eTable 4.** Characteristics of Safety-Net Hospitals Using Area -Level Definitions, 2022 **eTable 5.** Stability of Safety-Net Status Over Time Across Measures, Stratified by Number of Years

Supplement 2.
Data Sharing Statement
