# Supplementary material for: Variation, Overlap, and Stability in Defining Safety Net Hospitals
Source: JAMA Netw Open. 2025 Jul 30;8(7):e2523923. doi: 10.1001/jamanetworkopen.2025.23923 (PMC12311695; doi:10.1001/jamanetworkopen.2025.23923)
Supplement: Supplement 1. — eTable 1. Description of Safety-Net Hospital Definitions & Implications for Policy and Payment eTable 2. Distribution of Safety-Net Candidate Measures, 2022 eTable 3. Characteristics of Safety-Net Hospitals Using Hospital-Level Definitions, 2022 eTable 4. Characteristics of Safety-Net Hospitals Using Area -Level Definitions, 2022 eTable 5. Stability of Safety-Net Status Over Time Across Measures, Stratified by Number of Years [file jamanetwopen-e2523923-s001.pdf]

Supplemental Online Content

Chatterjee P, Liao JM, Amagai K, Zhao Y, Shirk T, Navathe AS. Variation, overlap, and stability in defining safety net hospitals. *JAMA Netw Open*. 2025;8(7):e2523923. doi:10.1001/jamanetworkopen.2025.23923

eTable 1. Description of Safety-Net Hospital Definitions & Implications for Policy and Payment

eTable 2. Distribution of Safety-Net Candidate Measures, 2022

eTable 3. Characteristics of Safety-Net Hospitals Using Hospital-Level Definitions, 2022

eTable 4. Characteristics of Safety-Net Hospitals Using Area -Level Definitions, 2022

eTable 5. Stability of Safety-Net Status Over Time Across Measures, Stratified by Number of Years

This supplemental material has been provided by the authors to give readers additional information about their work.

© 2025 Chatterjee P et al. *JAMA Network Open*

eTable 1: Description of Safety-Net Hospital Definitions & Implications for Policy and Payment

	Safety-net measure	Definition & policy relevance	Funding implications
Federal	Medicare Disproportionate Share Hospital (DSH) Index	Medicare DSH payments are provided to hospitals that treat high percentages of low-income Medicare beneficiaries. A hospital's eligibility and payments are determined by its DSH patient percentage, which considers the proportion of Medicare inpatient days attributable to patients eligible for both Medicare Part A and Supplemental Security Income, and the percentage of total inpatient days attributable to patients eligible for Medicaid but not Medicare Part A.	The criteria used to calculate the DSH patient percentage determine which hospitals qualify for these payments and the amount they receive. Total federal Medicare DSH payments are estimated at $14 billion in 2025.
	Medicare Safety Net Index (MSNI)	MSNI is an index for identifying hospitals that serve low-income Medicare beneficiaries. It is calculated as a composite of the share of dual eligibles or those who qualify for the low-income subsidy, hospital's provision of uncompensated care, and the hospital's Medicare share.	Though not officially used in payment policy yet, MedPAC has proposed using MSNI to reallocate Medicare DSH payments and other supplemental payments to better target safety net hospital recipients.
	Dual & low-income subsidy (DLIS) inpatient day share	DLIS inpatient day share reflects the share of inpatients who are either dual eligibles or eligible for enrollment in Medicare's low-income subsidy program, which targets low-income individuals for support with prescription drug payments.	The DLIS share is a component of the MSNI and influences safety net hospital payment through this mechanism.
	Medicaid inpatient day share	Medicaid inpatient share reflects the share of inpatients insured by the Medicaid program. It is used in policy decisions related to the allocation of Medicaid DSH payments, provider taxes, and other supplemental payments designed to support safety net care delivery.	Medicaid DSH payments are allocated to hospitals that disproportionately serve Medicaid and uninsured individuals. States have discretion in defining which hospitals qualify, leading to variations across states. Total Medicaid DSH payments, which are jointly financed by state and federal governments, were $19 billion in FY 2021. Over $50 billion in supplemental payments (of which Medicaid DSH is one type) were allocated to hospitals in 2022.
	Teaching status	Teaching hospitals (major and minor) provide educational services and opportunities to medical trainees. They are often located in large, urban areas and serve high volumes of low-income patients and patients insured by Medicaid.	Teaching hospitals receive financial support for uncompensated care and clinical service to low-income populations in the form of Medicare and Medicaid DSH payments, 340B payments, and other supplemental funds. They are typically exempt from federal taxes, and some state and local taxes.
	Area Deprivation Index (ADI)	ADI is a census tract-level measure of socioeconomic disadvantage, components of which (low-income,	CMS has used the ADI to adjust payment rates and quality scores in certain payment models (ACO REACH, MSSP; under

© 2025 Chatterjee P et al. *JAMA Network Open*

		unemployment, low levels of education, poor housing quality) are disproportionately prevalent among safety net patient populations.	consideration for Medicare Advantage Star Ratings) to account for providers serving substantial numbers of low-income and disadvantaged patient populations. It has not been deployed specifically in safety net hospital financing mechanisms.
	Social Vulnerability Index (SVI)	SVI is a census tract-level measure of demographic and socioeconomic factors to estimate the impact of emergency events (such as natural or human-caused disasters, or disease outbreaks)	SVI was used by the CDC during the COVID-19 pandemic to allocate health care resources to communities at disproportionate risk for infection, including those with limited medical capacity and resources, as well as racial and ethnic minority communities which had higher risk for infection in certain settings. It has not been deployed specifically in safety net hospital financing mechanisms.
State/ local	Public ownership	Publicly owned (non-federal) hospitals are typically owned by local, municipal, and/or state governments and have historically been considered safety net hospitals.	Public hospitals rely on local, municipal, and state governments for financing through multiple mechanisms, some of which are related to Medicaid financing (such as provider taxes).
	Uncompensated care share	Hospitals that serve low-income, uninsured, and underinsured patients often provide uncompensated care, defined as the sum of charity care and bad debt.	High levels of uncompensated care can introduce financial challenges for hospitals and restrict their viability. Measures of uncompensated care are incorporated into the MSNI and the allocation of Medicaid DSH payments. Several states have also begun to develop funding pools with the primary purpose of ensuring the financial viability of safety net hospitals.
	Operating margin	Hospitals that serve low-income, uninsured, and underinsured patients often provide uncompensated care which can be associated with lower operating margins.	Operating margins are a marker of a hospital's overall finances and have implications for its viability. Several states have begun to develop funding pools with the primary purpose of ensuring the financial viability of safety net hospitals.
	Medicaid inpatient day share	As above	As above
	Teaching	As above	As above
Other	Percent Black population	Patients from racial minority groups are more likely to be represented among low-income populations served by safety net hospitals.	Medicare, Medicaid, and other payers have deployed efforts to measures health disparities across racial minority and non-minority populations and incentivized reductions in disparities through payment.
	Percent Hispanic population	Patients from ethnic minority groups are more likely to be represented among low-income populations served by safety net hospitals.	Medicare, Medicaid, and other payers have deployed efforts to measure health disparities across ethnic minority and non-minority populations and incentivized reductions in disparities through payment.

© 2025 Chatterjee P et al. *JAMA Network Open*

eTable 2: Distribution of Safety-net Candidate Measures, 2022

	Min	P25	Median	Mean	P75	Max
**Medicare DSH**	0	0	21.5	21.2	33.1	173.6
**Medicaid Inpatient Day Share**	0	6.8	17.1	18.3	25.9	100.0
**MSNI**	0	54.2	64.6	66.0	76.5	173.3
**DLIS Inpatient Day Share**	0	5.3	8.5	9.6	12.6	120
**Medicare Inpatient Day Share**	0	43.3	52.9	51.6	62.2	100
**Uncompensated Care Share**	0	3.2	5.9	7.2	9.5	95.1
**Operating Margin**	-4.3	-0.1	0	0	0.1	3.4
**ADI**	1.0	43.0	67.0	61.7	84.0	100.0
**SVI**	0	0.4	0.6	0.6	0.8	1.0
**% Black**	0	1.0	3.5	10.6	12.5	95.9
**% Hispanic**	0	3.1	6.9	13.8	16.8	97.3

© 2025 Chatterjee P et al. *JAMA Network Open*

eTable 3: Characteristics of Safety-Net Hospitals using Hospital-Level Definitions, 2022

	All Hospitals		DSH Index		Medicare Inpatient Days Share		MSNI*		DLIS Inpatient Days Share		Medicaid Inpatient Days Share		Teaching		Public		Operating Margin		Uncompensated Care Share*	
**Total**	4,531		1,133		1,133		1,133		1,133		1,141		1,326		992		1,106		1,141	
**Non-Metro**	1,837	41%	231	20%	673	59%	581	51%	599	53%	327	29%	127	10%	644	65%	466	42%	551	48%
**Beds**																				
0-99	2,558	56%	299	26%	883	78%	715	63%	669	59%	381	33%	146	11%	761	77%	635	57%	768	67%
100-299	1,256	28%	481	42%	199	18%	313	28%	373	33%	428	38%	632	48%	116	12%	327	30%	278	24%
>= 300	661	15%	353	31%	41	4%	91	8%	84	7%	330	29%	547	41%	104	10%	131	12%	86	8%
**Teaching hospital**	1,326	29%	621	55%	165	15%	286	25%	295	26%	576	50%	1,326	100%	167	17%	324	29%	247	22%
**Ownership**																				
Public	992	22%	223	20%	308	27%	344	30%	248	22%	234	21%	167	13%	992	100%	292	26%	283	25%
Private, nonprofit	2,751	61%	646	57%	670	59%	563	50%	707	62%	691	61%	940	71%	0	0%	661	60%	687	60%
Private, for profit	788	17%	264	23%	155	14%	226	20%	178	16%	216	19%	219	17%	0	0%	153	14%	171	15%
**Region**																				
Northeast	543	12%	157	14%	133	12%	160	14%	198	17%	137	12%	289	22%	26	3%	186	17%	137	12%
Midwest	1,360	30%	177	16%	512	45%	217	19%	274	24%	342	30%	318	24%	287	29%	302	27%	342	30%
South	1,703	38%	466	41%	367	32%	503	44%	406	36%	429	38%	440	33%	441	44%	380	34%	429	38%
West	925	20%	333	29%	121	11%	253	22%	255	23%	233	20%	279	21%	238	24%	238	22%	233	20%
**DSH Index,** mean (sd)	21.2 (20.8)		48.4 (16.1)		8.5 (11.7)		27.6 (27.6)		26.7 (21.4)		41.8 (21.3)		35.0 (17.9)		17.2 (23.7)		24.0 (22.1)		21.7 (22.9)	

© 2025 Chatterjee P et al. *JAMA Network Open*

**Medicaid Inpatient Days Share**, mean (sd)	18.3 (14.2)	34.6 (12.4)	8.4 (7.0)	20.3 (18.1)	20.4 (13.2)	35.2 (13.1)	26.0 (12.4)	16.4 (17.2)	19.4 (15.2)	18.9 (15.5)
**Uncompensated Care Share**, mean (sd)	7.2 (6.6)	8.4 (8.7)	7.0 (5.8)	11.2 (10.2)	7.1 (5.6)	8.0 (8.4)	7.0 (7.2)	8.6 (9.4)	8.4 (6.5)	14.1 (9.1)
**Operating Margin**, mean (sd)	0.0 (0.1)	0.0 (0.1)	0.0 (0.1)	0.0 (0.1)	0.0 (0.1)	0.0 (0.1)	0.0 (0.1)	0.0 (0.1)	-0.2 (0.1)	0.0 (0.1)
**Median Household Income ($)**, mean (sd)	68,966.90 (26,101.50)	66,828.50 (27,571.40)	65,113.02 (22,332.15)	60,239.20 (23,517.00)	63,963.40 (24,461.80)	63,488.10 (22,574.60)	74,261.10 (32,196.00)	61,248.10 (20,483.40)	67,426.40 (25,849.20)	66,248.20 (24,666.50)
**Unemployment Rate**, mean (sd)	5.3 (3.1)	6.6 (3.3)	4.7 (2.9)	6.5 (3.7)	6.0 (3.3)	6.1 (3.5)	5.9 (2.9)	5.6 (4.3)	5.5 (2.9)	5.4 (2.8)
**Percent High School Graduate,** mean (sd)	88.9 (6.5)	86.6 (8.0)	89.2 (5.6)	84.7 (7.1)	87.1 (6.6)	87.4 (7.8)	89.4 (7.3)	87.0 (7.1)	88.2 (6.9)	87.6 (6.5)
**ADI,** mean (sd)	61.7 (26.8)	53.9 (30.4)	70.6 (22.0)	64.5 (29.1)	61.8 (28.4)	61.6 (26.5)	51.1 (29.6)	69.2 (241.1)	63.0 (27.6)	63.8 (26.7)
**SVI,** mean (sd)	0.6 (0.2)	0.7 (0.2)	0.6 (0.2)	0.7 (0.2)	0.7 (0.2)	0.7 (0.2)	0.6 (0.3)	0.7 (0.2)	0.6 (0.2)	0.7 (0.2)
**% Black,** mean (sd)	10.6 (16.2)	17.0 (20.6)	7.5 (14.3)	15.1 (21.3)	11.7 (18.5)	15.9 (20.2)	15.5 (18.6)	11.9 (19.1)	12.4 (18.9)	10.7 (17.0)

© 2025 Chatterjee P et al. *JAMA Network Open*

**% Hisp,** mean (sd)	13.8 (17.3)		22.6 (23.6)		8.9 (12.6)		19.8 (24.1)		14.4 (19.3)		18.2 (21.7)		17.5 (19.0)		14.3 (18.2)		14.9 (18.7)		15.6 (19.2)	
**Medicare Inpatient Days Share,** mean (sd)	51.6 (17.0)		42.5 (12.9)		71.1 (7.4)		55.2 (18.7)		55.9 (14.1)		41.1 (13.6)		48.9 (12.8)		51.6 (20.1)		51.5 (18.1)		51.0 (17.5)	
**MSNI,** mean (sd)	61.1 (19.1)		69.6 (22.3)		68.4 (14.8)		85.6 (15.5)		73.8 (17.3)		63.6 (21.6)		61.5 (19.2)		66.0 (21.2)		66.2 (19.7)		71.9 (21.1)	
**DLIS Inpatient Days Share**	9.6 (6.7)		11.8 (6.9)		11 (8.2)		14.0 (8.9)		18.3 (6.7)		10.0 (5.8)		9.8 (5.6)		9.9 (7.9)		10.7 (6.9)		10.0 (6.9)	
**Location in Medicaid expansion states**	3071	68%	832	73%	750	66%	752	66%	862	76%	773	68%	990	75%	572	58%	758	69%	773	68%

© 2025 Chatterjee P et al. *JAMA Network Open*

eTable 4: Characteristics of Safety-Net Hospitals using Area -Level Definitions, 2022

	Top Quartile ADI		Top Quartile SVI		Top Quartile Percent Black population		Top Quartile Percent Hispanic population	
**Total**	1,117		1,057		1,076		1,074	
**Non-Metro**	703	63%	428	40%	276	26%	291	27%
**Beds**								
0-99	787	70%	540	51%	429	40%	483	45%
100-299	215	19%	314	30%	383	36%	378	35%
>= 300	103	9%	191	18%	247	23%	196	18%
**Teaching hospital**	222	20%	342	32%	461	43%	394	37%
**Ownership**								
Public	314	28%	221	21%	255	24%	239	22%
Private, nonprofit	660	59%	569	54%	575	53%	539	50%
Private, for profit	143	13%	267	25%	246	23%	296	28%
**Region**								
Northeast	93	8%	109	10%	120	11%	108	10%
Midwest	489	44%	183	17%	169	16%	101	9%
South	459	41%	514	49%	751	70%	439	41%
West	76	7%	251	24%	36	3%	426	40%
**DSH Index,** mean (sd)	17.3 (20.0)		27.5 (24.4)		29.4 (20.7)		28.5 (24.4)	
**Medicaid Inpatient Days Share**, mean (sd)	15.9 (14.0)		22.0 (16.0)		21.7 (15.0)		22.3 (16.0)	
**Uncompensated Care Share**, mean (sd)	7.6 (7.0)		8.5 (8.0)		7.9 (8.0)		9.5 (8.0)	
**Operating Margin**, mean (sd)	0.0 (12.0)		0.0 (12.0)		0.0 (12.0)		0.0 (13.0)	
**Median Household Income ($)**, mean (sd)	54876.0 (12539.0)		57471.0 (17945.0)		58210.5 (21355.0)		70304.5 (23686.0)	
**Unemployment Rate**, mean (sd)	5.6 (3.0)		6.7 (3.0)		6.8 (3.0)		5.8 (3.0)	
**Percent High School Graduate,** mean (sd)	87.0 (6.0)		84.8 (7.0)		86.3 (7.0)		84.4 (8.0)	

© 2025 Chatterjee P et al. *JAMA Network Open*

**ADI,** mean (sd)	87.2 (7.0)		71.5 (24.0)		66.6 (26.0)		52.8 (29.0)	
**SVI,** mean (sd)	0.7 (19.0)		0.9 (7.0)		0.7 (24.0)		0.7 (22.0)	
**% Black population,** mean (sd)	12.4 (19.0)		17.1 (21.0)		33.0 (19.0)		9.7 (12.0)	
**% Hispanic population,** mean (sd)	10.4 (15.0)		20.8 (22.0)		12.9 (14.0)		38.0 (19.0)	
**Medicare Inpatient Days Share,** mean (sd)	55.0 (18.0)		49.7 (17.0)		48.7 (17.0)		47.1 (16.0)	
**MSNI,** mean (sd)	65.2 (17.0)		68.6 (21.0)		64.6 (22.0)		66.3 (23.0)	
**Medicaid expansion status**	17	20%	28	24%	29	21%	29	24%

© 2025 Chatterjee P et al. *JAMA Network Open*

eTable 5: Stability of Safety-Net Status Over Time Across Measures, Stratified by Number of Years

	Total SNHs in 2014, 2018, or 2022	Only SNH 2014		Only SNH 2018		Only SNH 2022		SNH in 2014, 2018		SNH in 2018, 2022		SNH in 2014, 2022		SNH in 2014, 2018, 2022	
	N	N	%	N	%	N	%	N	%	N	%	N	%	N	%
**Medicare DSH**	1,358	94	7%	51	4%	142	10%	104	8%	108	8%	50	4%	809	60%
**Medicare Inpatient Days Share**	1,504	141	9%	89	6%	254	17%	222	15%	117	8%	57	4%	624	41%
**MSNI**	1,411	123	9%	76	5%	216	15%	152	11%	126	9%	45	3%	673	48%
**DLIS Inpatient Days Share**	1,725	309	18%	149	9%	317	18%	176	10%	274	16%	64	4%	436	25%
**Medicaid Inpatient Days Share**	1,499	166	11%	96	6%	177	12%	130	9%	138	9%	73	5%	719	48%
**Teaching**	1,354	15	1%	9	1%	150	11%	25	2%	147	11%	8	1%	1,000	74%
**Public Hospital**	1,043	54	5%	4	0%	36	3%	56	5%	29	3%	2	0%	862	83%
**Operating Margin**	1,796	306	17%	238	13%	431	24%	235	13%	204	11%	119	7%	263	15%
**Uncompensated Care Share**	1,823	339	19%	234	13%	328	18%	209	11%	221	12%	130	7%	362	20%
**ADI**	1,175	5	0%	2	0%	235	20%	146	12%	1	0%	1	0%	785	67%
**SVI**	1,291	96	7%	98	8%	158	12%	107	8%	104	8%	83	6%	645	50%
**% Black population**	1,084	29	3%	16	1%	50	5%	41	4%	40	4%	20	2%	888	82%
**% Hispanic population**	1,067	36	3%	21	2%	39	4%	37	3%	43	4%	27	3%	864	81%

© 2025 Chatterjee P et al. *JAMA Network Open*
