# Supplementary material for: Variation, Overlap, and Stability in Defining Safety Net Hospitals
Source: JAMA Netw Open. 2025 Jul 30;8(7):e2523923. doi: 10.1001/jamanetworkopen.2025.23923 (PMC12311695; doi:10.1001/jamanetworkopen.2025.23923)
Supplement: Supplement 2. — Data Sharing Statement [file jamanetwopen-e2523923-s002.pdf]

Data Sharing Statement

Chatterjee. Variation, Overlap, and Stability in Defining Safety Net Hospitals. *JAMA Netw Open*. Published July 30, 2025. doi:10.1001/jamanetworkopen.2025.23923

Data
Data available: No

Additional Information
Explanation for why data not available: This study uses Medicare claims data which are available through separate data use agreements with CMS.
